# ‘*Candidatus* Phytoplasma mali’ SAP11-Like protein modulates expression of genes involved in energy production, photosynthesis, and defense in *Nicotiana occidentalis* leaves

**DOI:** 10.1186/s12870-024-05087-4

**Published:** 2024-05-13

**Authors:** Cecilia Mittelberger, Mirko Moser, Bettina Hause, Katrin Janik

**Affiliations:** 1 Molecular Biology and Microbiology, Group of Functional Genomics, Laimburg Research Centre, Pfatten (Vadena), South Tyrol 39051 Italy; 2 Department of Genomics and Biology of Fruit Crops, Research and Innovation Centre, Fondazione Edmund Mach, San Michele All’Adige, Trentino, 39098 Italy; 3 Department of Cell and Metabolic Biology, Leibniz Institute of Plant Biochemistry, 06120 Halle (Saale), Saxony-Anhalt, Germany

**Keywords:** Apple proliferation, Plant defense, RNA-seq, SAP11

## Abstract

**Background:**

‘*Candidatus* Phytoplasma mali’, the causal agent of apple proliferation disease, exerts influence on its host plant through various effector proteins, including SAP11_CaPm_ which interacts with different TEOSINTE BRANCHED1/ CYCLOIDEA/ PROLIFERATING CELL FACTOR 1 and 2 (TCP) transcription factors. This study examines the transcriptional response of the plant upon early expression of *SAP11*_*CaPm*_. For that purpose, leaves of *Nicotiana occidentalis* H.-M. Wheeler were Agrobacterium-infiltrated to induce transient expression of *SAP11*_*CaPm*_ and changes in the transcriptome were recorded until 5 days post infiltration.

**Results:**

The RNA-seq analysis revealed that presence of SAP11_CaPm_ in leaves leads to downregulation of genes involved in defense response and related to photosynthetic processes, while expression of genes involved in energy production was enhanced.

**Conclusions:**

The results indicate that early *SAP11*_*CaPm*_ expression might be important for the colonization of the host plant since phytoplasmas lack many metabolic genes and are thus dependent on metabolites from their host plant.

**Supplementary Information:**

The online version contains supplementary material available at 10.1186/s12870-024-05087-4.

## Background

*‘Candidatus* Phytoplasma mali*’* (‘*Ca*. P. mali’) is a plant pathogen, associated to Apple proliferation disease in apple (*Malus* x *domestica* Borkh.). This cell wall-less bacterium belongs to the class of Mollicutes and has one of the smallest genomes among all so far fully sequenced phytoplasma species [1]. Phytoplasmas reside in the plant phloem and are transmitted by phloem sucking psyllids. ‘*Ca*. P. mali’ manipulates its host plant by secreting effector proteins via a sec-dependent secretion system [2]. Several effector proteins are known from different phytoplasma [3]. So far, in ‘*Ca*. P. mali’ four effector proteins, namely SAP11_CaPm_ [4], PME2 [5], PM19_00185 [6] and SAP05_CaPm_ [7], as well as the virulence factor AAA + ATPase AP460 [8] have been identified as host manipulating factors. While little or nothing is known about PME2’s, SAP05_CaPm_´s and PM19_00185´s function in apple trees, the potential function of the ‘*Ca*. P. mali’ SAP11, homolog of SAP11_AYWB_ (from ‘*Candidatus* Phytoplasma asteris’), has been also described in apple [4, 9–11]. SAP11_AYWB_ binds and destabilizes three different TCP (TEOSINTE BRANCHED1/ CYCLOIDEA/ PROLIFERATING CELL FACTOR 1 and 2) transcription factors and is involved in the development of different symptoms [12, 13]. In contrast to SAP11_AYWB_, SAP11_CaPm_ localizes not only to the cell nucleus, but also to the cytoplasm [14]. However, it has been shown that it binds (similar as SAP11_AYWB_) two class II CIN-like TCPs, namely MdTCP4a (orthologue to AtTCP4) and MdTCP13a (orthologue to AtTCP13) [4], formerly known as MdTCP25 and MdTCP24 respectively [15] as well as to the class II CYC/TB1 TCP MdTCP18a (orthologue to AtTCP18) (formerly known as MdTCP16 as described in [16]). SAP11_CaPm_-binding to its TCP-interaction partners causes severe growth aberrations, early bud break and hormonal disbalance within the plant [14]. Effector binding of MdTCP4a and MdTCP13a is supposed to be at the basis of the changes in jasmonate (JA) and abscisic acid (ABA) levels observed in infected plants and might be the reason for the development of late flowers, leaf reddening and altered root architecture [4]. The binding of MdTCP18a is supposed to counteract the *MdTCP18a* upregulation in infected plants, leading to an early bud break and uncontrolled shoot outgrowth [16].

The stable overexpression of *SAP11*_*AYWB*_ in *Arabidopsis* plants resulted in a total of 59 upregulated and 104 downregulated genes as revealed by RNA-seq [17]. From the 59 upregulated genes, 18 genes were functionally annotated as inorganic phosphorus (P_i_) starvation-induced genes. In the group of downregulated genes, *LIPOXYGENASE2* (*LOX2*), a gene encoding an enzyme involved in JA biosynthesis, and *PATHOGENESIS-RELATED GENE1 (PR1)* and *ELICITOR-ACTIVATED GENE3-1 (ELI3-1)*, two salicylic acid (SA) responsive genes, were found. This indicates that SAP11_AYWB_ suppresses the defense response while enhancing bacterial growth in *Arabidopsis* plants. In addition, it has been shown that defense response to insect vectors is also reduced in SAP11_AYWB_ overexpressing *Arabidopsis* plants [18]. *Nicotiana occidentalis* H.-M. Wheeler plants directly infected with ‘*Ca*. P. mali’ showed 157 proteins with an increased and 173 with a decreased expression compared to healthy plants. This highlights the fact that a single effector, such as SAP11, is only partially involved in the pathogen induced transcriptional changes [19]. The proteins encoded by genes with an increased expression comprised mainly the alpha-linolenic acid synthesis, while those with downregulation were involved in porphyrin and chlorophyll metabolism [19]. This was in line with increased JA levels and leaf yellowing of infected plants.

Even though such studies help to understand possible functions of SAP11_CaPm_, only little is known so far about the very early role of SAP11_CaPm_ during early infection of plants with ‘*Ca*. P. mali’. Thus, the aim of this study was to gain a better understanding of the transcriptional changes that occur in the plant host during early occurrence of the effector protein. Infiltration RNA-seq [20] was used to unravel expression networks and effector function in healthy plants upon expression of *SAP11*_*CaPm*_. Moreover, *N. occidentalis* H.-M. Wheeler was chosen since it has been described as the appropriate model plant to study ‘*Ca*. P. mali’ effector functions [10, 19, 21–23]. Therefore, the gene encoding the effector protein SAP11_CaPm_ was transiently expressed by agroinfiltration in *N. occidentalis* H.-M. Wheeler leaves and differential gene expression was analyzed until 5 days post infiltration in the respective leaf tissue. Transcriptional changes in the infiltrated leaves revealed that SAP11_CaPm_ affects mainly genes involved in defense responses, photosynthesis, and pathways involved in the production of energy equivalents at early time points of its occurrence in the cells.

## Methods

### Plant material and agroinfiltration

*Nicotiana occidentalis* H.-M. Wheeler seeds were kindly provided by Kajohn Boonrod from RLP AgroScience GmbH, Neustadt, Germany [10, 21]. Seedlings were grown in a plant growth chamber (Percival AR22L, Percival Scientific, Perry, IA, USA) under long photoperiod conditions (16 h/8 h, 24 °C/22°C, 70% rH). Four to five-week-old plants were used for agroinfiltration.

For agroinfiltration the coding sequence of the mature SAP11_CaPm_ effector protein from ‘*Ca*. P. mali’ strain STAA (Accession: KM501063) was subcloned into the GreenGate-entry module pGGC00 [24] using the primer pair ATP00189pP_Cfw (AACAGGTCTCAGGCTCCATGTCTCCTCCTAAAAAAGATTCTA) / ATP00189pP_Drv (AACAGGTCTCACTGATTTTTTTCCTTTGTCTTTATTGTTA).

Transformation constructs coding for SAP11_CaPm_:GFP under the control of CaMV *35 S* promoter and flanked by the RBCS terminator and a plant kanamycin resistance marker were assembled using modules from the GreenGate-kit [24]. In detail, a GreenGate reaction containing 150 ng pGGA004 (*p35S*), 150 ng pGGB003 (B-dummy), 150 ng pGGC000- SAP11_CaPm_, 150 ng pGGD001 (linker-GFP), 150 ng pGGE001 (*tRBCS*), 150 ng pGGF007 (*pNOS::KanR: tNOS*), and 100 ng pGGZ001 (empty destination vector) was combined in a total volume of 15 µL. For the GreenGate reaction 1.5 µL 10× CutSmart Buffer (New England Biolab, Ipswich, MA, USA), 1.5 µL ATP (10 mM), 1.0 µL T4 DNA Ligase (5 u/µL) (Thermo Fisher Scientific, Waltham, MA, USA), and 1.0 µL BsaI-HF®v2 (20,000 u/mL) (New England Biolab, Ipswich, MA, USA) were added to the module-mixture, and 30 cycles at 37 °C and at 16 °C for 2 in each, followed by 50 °C for 5 min and 80 °C for 5 min were performed. Subsequently, 5 µL of the reaction mixture were used for heat-shock transformation of ccdB-sensitive One Shot® TOP10 chemically competent *E. coli* (Invitrogen, Carlsbad, CA, USA). A second vector containing only the GFP gene fused to a nuclear localization signal was assembled, using the pGGC012 module (GFP-NLS). The correctness of the assembled plant expression vectors was confirmed by sequencing.

The validated GreenGate expression vectors, *35 S::SAP11*_*CaPm*_:*GFP* and *35 S::GFP-NLS* were transferred together with *pSOUP* helper plasmid into electrocompetent *A. tumefaciens* strain EHA105. The *A. tumefaciens* clones were cultured for 2 days at 28 °C in selective LB medium. For infiltration 0.5 OD/mL were resuspended in infiltration medium (10 mM MgCl2, 10 mM MES, 200 µM acetosyringone, pH 5.7), regenerated for 4 h at 28 °C and infiltrated with a blunt syringe into three leaves from four- to five-week-old *N. occidentalis* H.-M. Wheeler plants. Six plants were infiltrated with the effector expressing *35 S::SAP11*_*CaPm*_:*GFP* construct and six plants were infiltrated with *35 S::GFP-NLS* serving as controls. Additional six plants were not infiltrated and used as non-infiltrated controls. The infiltrated area was marked with a pen on the adaxial leaf side and six leaf discs with a size of 1 cm² (two/infiltrated area) were excised immediately after infiltration from one plant of each variant. Leaf disc excision was repeated on different plants in a 24-h-rhythm. Leaf discs were immediately flash frozen in liquid nitrogen. Infiltration and leaf disc sampling were repeated with three independent plant sets, grown at three different time points.

### cDNA library construction and sequencing

A total of 36 leaf discs samples of plants from all three treatments for the timepoints 0 h, 24 h, 72 h and 120 h were sent on dry ice for RNA extraction, library preparation and sequencing to StarSEQ (Mainz, Germany). Additionally, 15 samples were prepared from different growth stages of untreated *N. occidentalis* H.-M. Wheeler plants and send on dry ice to StarSEQ. RNA of those samples together with RNA of the 36 leaf disc samples was pooled and used for the de novo transcriptome assembly.

The stranded RNA sequencing library was prepared using the NEBNext Ultra II Directional RNA Library Prep Kit for Illumina (New England Biolab, Ipswich, MA, USA). The library for the de novo transcriptome assembly was sequenced on a Illumnia NextSeq500 platform in 2 × 150 nt paired-end mode. The libraries of the 36 leaf samples were sequenced on the same platform in 1 × 75 nt single-end mode.

Quality assessment of the reads was performed using the FASTQC tool [25].

Adapter trimming of paired end reads was done with the FASTQ Toolkit (BaseSpace Labs) retaining reads with a minimum read length of 32 nt.

### De novo transcriptome assembly of *Nicotiana occidentalis*

The workflow for the de novo assembly of the transcriptome and the RNA-seq analysis was carried out as follows: First, all adapter trimmed paired end reads were quality trimmed with a sliding window of 4 nt, a minimum phred quality score of 20 and a minimal read length of 75 nt using the tool Trimmomatic v.0.36 [26]. The de novo assembly was then performed in strand-specific mode using Trinity v. 2.9 [27]. The whole de novo assembled transcriptome was first annotated using Trinotate v.3.2.0 [28] with the help of Transdecoder v.5.5.0 [29] to estimate all possible coding regions. Since the annotation contained several transcripts not belonging to *Nicotiana*, the whole transcriptome was decontaminated using the MCSC Decontamination method [30] filtering for transcripts belonging to the order of *Solanales.* The remaining decontaminated transcriptome was reannotated with Trinotate v.3.2.1 using homology search to SwissProt sequence database with Blast 2.12.0+ [31], to PFAM database for protein domain identification with HMMER (hmmer v.3.3.2) [32] and for the prediction of a signal peptide with SignalP v.5.0.b [33] and of a transmembrane domain with tmhmm v.2.0c [34]. The annotated transcripts were visualized using the build in TrinotateWeb tool.

### Differential expression analysis, go enrichment analysis

Transcripts were quantified using Trinity v.2.11.0 build in Salmon (v.1.4.0) [35] pipeline. Selection of differentially expressed transcripts (DETs) was afterwards performed with the Trinity v.2.11.0 build in DESeq2 pipeline [36], where parameters for filtering are set to > 4-fold change and a false discovery rate (FDR) < 0.001.

Lists of DETs were annotated by homology search with Blast 2.12.0+ [31] against standard nucleotide collection database (nt) with an e-value cut off set to 0.001.

The lists of DETs were further analyzed and subset by Venn diagrams using jvenn [37].

Gene ontology (GO) assignments were first extracted from Trinotate output and then all lists of up and downregulated DETs and the Venn subsets were functional enriched with the Trinity v2.11.0 build in GOseq [38] pipeline, using the *de-novo* assembled and decontaminated *N. occidentalis* H.-M. Wheeler transcriptome as background.

The functional enrichment was visualized using RStudio 2022.07.1 (RStudio, PBC) with R v4.2.0 [39] and the Bioconductor packages goseq v.1.48.0 [40] and rrvgo v.1.8.0 [41].

For a detailed analysis of enriched transcripts, the GO assignments were filtered with a script for defence or stress related terms and phytohormone related terms. In detail, files with GO assignments were searched using strings for defence/stress (“stress”, “defence”, “immune”) and for phytohormone related terms (“salicylic”, “auxin”, “gibberel*”, “jasmonic”, “ethylene”, “cytokinine”, “abscisic”, “brassinosteroi*”; asterisks in the term indicate placeholder for any letter).

For further functional characterization of up- and downregulated transcripts the different subsets were analyzed with STRING database v.12.0 [42] using the whole *N. tabacum* L. genome as background for network analysis. In detail the protein sequences of DETs were uploaded to the multiple sequences search interface, annotated with STRING by homology search within the *N. tabacum* L. genome and network was visualized with protein interactions based on functional and physical protein associations. The network was then clustered with MCL (Markov Cluster Algorithm) [43] clustering using an inflation parameter of 4. Enriched gene ontologies and KEGG pathways of the biggest cluster were downloaded.

### RNA extraction, cDNA synthesis and qPCR

For validation of DETs, the agroinfiltration approach was repeated with a new set of plants, using three biological replicates. The excised leaf discs were immediately flash frozen in liquid nitrogen, grinded using a mortar and pistil, and 100 mg of frozen leaf powder was used for RNA extraction with Spectrum™ Plant Total RNA Kit (Merck, Darmstadt, Germany) following protocol A of the manual. RNA concentration was measured with a spectrophotometer (Implen N60). Using 2 µg of RNA, genomic DNA removal and cDNA synthesis was performed with SuperScript™ IV VILO™ Master Mix with ezDNase™ enzyme.

To find suitable and stable expressed reference genes, primer pairs for NbPP2a, NbNQO, NbGAPDH and NbEF1a, identified as reference genes in *N. benthamiana* Domin [44] as well as the endogenous universal qPCR control UNI28S [45] were selected. They *in-silico* matched (tested with Geneious R11.1.5) to transcripts within the *de-novo* assembled *N. occidentalis* H.-M. Wheeler transcriptome and were thus tested in a qPCR assay. The qPCR data of all candidates were analyzed by RefFinder [46] and the two most stable genes, *PP2a* and *NQO* were used as reference genes.

Diluted cDNA was used for qPCR assays with three technical replicates, using SYBR chemistry. In detail, 2 µL of template were used in a 10 µL reaction mixture containing 5 µL 2x SYBR FAST qPCR Kit Master Mix (Kapa Biosystems), 2.6 µL nuclease-free water and 0.20 µL each of forward and reverse primer (10 µM). All qPCR reactions were run on a CFX384 Touch Real-Time PCR Detection system, using the following conditions: initial denaturation at 95 °C for 20 s; 35 cycles of 95 °C for 3 s and 60 °C for 30 s; and a melting curve ramp from 65 to 95 °C at increments of 0.5 °C every 5 s. Melting curve analysis was performed to confirm the generation of the correct amplicon. Specific melting curves for every target amplified by SYBR green qPCR are provided in Additional file 1.

To determine qPCR efficiency of the respective target together with each qPCR run a five-point serial dilution of *N. occidentalis* H.-M. Wheeler cDNA (1:10, 1:20, 1:50, 1:100, 1:200) was analyzed. As an additional quality control of qPCR, a three-point serial dilution (1:10, 1:50, 1:100) was analyzed, amplifying the reference genes *NbNQO* and *NbPP2a*. Data analysis was performed using CFX Manager Software (Bio-Rad) and RStudio 2022.07.1 (RStudio, PBC) with R v4.2.0 [39] using the MCMC qPCR package (v.1.2.4) [47] applying an informed model.

## Results

### Expression of SAP11_CaPm_ in *Nicotiana occidentalis*

**Fig. 1:**
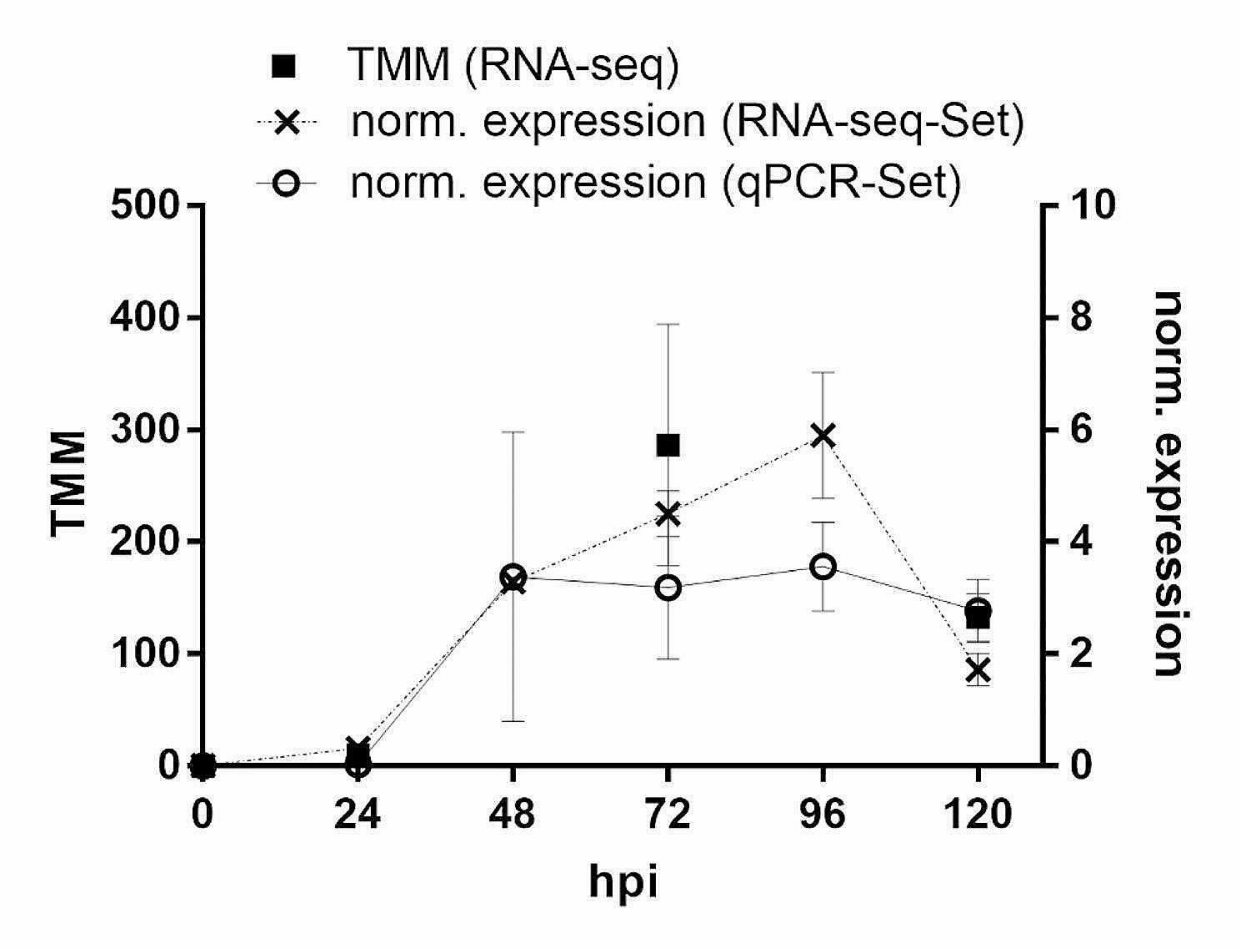
Expression of SAP11_CaPm_ in leaf samples analyzed by RNA-seq and qPCR. Beside the RNA-seq sample set a second independent sample set was analyzed only by qPCR. The RNA-seq set was analyzed at 0 h, 24 h, 72 h and 120 h, while the qPCR set was analyzed at every timepoint. Data represent the mean +/- SEM of 3 biological replicates. TMM = trimmed mean of M values [48]

To analyze early effects of *SAP11*_*CaPm*_ expression on the transcriptome of *N. occidentalis* H.-M. Wheeler, leaves were infiltrated with *A. tumefaciens* harboring a construct encoding SAP11_CaPm_ fused to GFP. As controls, infiltration with nuclear localized GFP (GFP-NLS) and non-infiltrated plants were used. Samples were taken every 24 h up to 120 h and subjected to RNA-seq. To verify the expression of *SAP11*_*CaPm*_, leaf samples later used for RNA-seq as well as leaf samples from a second independent experiment, were analyzed by RT-qPCR (Fig. 1). Within 24 h after infiltration, the first transcript accumulation was detectable. In the leaf samples set used for RNA-seq, the expression reached its maximum 96 h after infiltration and decreased 120 h post infiltration. Coherently, the analysis on the number of reads obtained by RNA-seq and indicative for expression of *SAP11*_*CaPm*_ showed this kinetics. In the second independent leaf set *SAP11*_*CaPm*_ expression was stable between 48 h and 96 h after infiltration and dropped only slightly after 120 h.

**Fig. 2:**
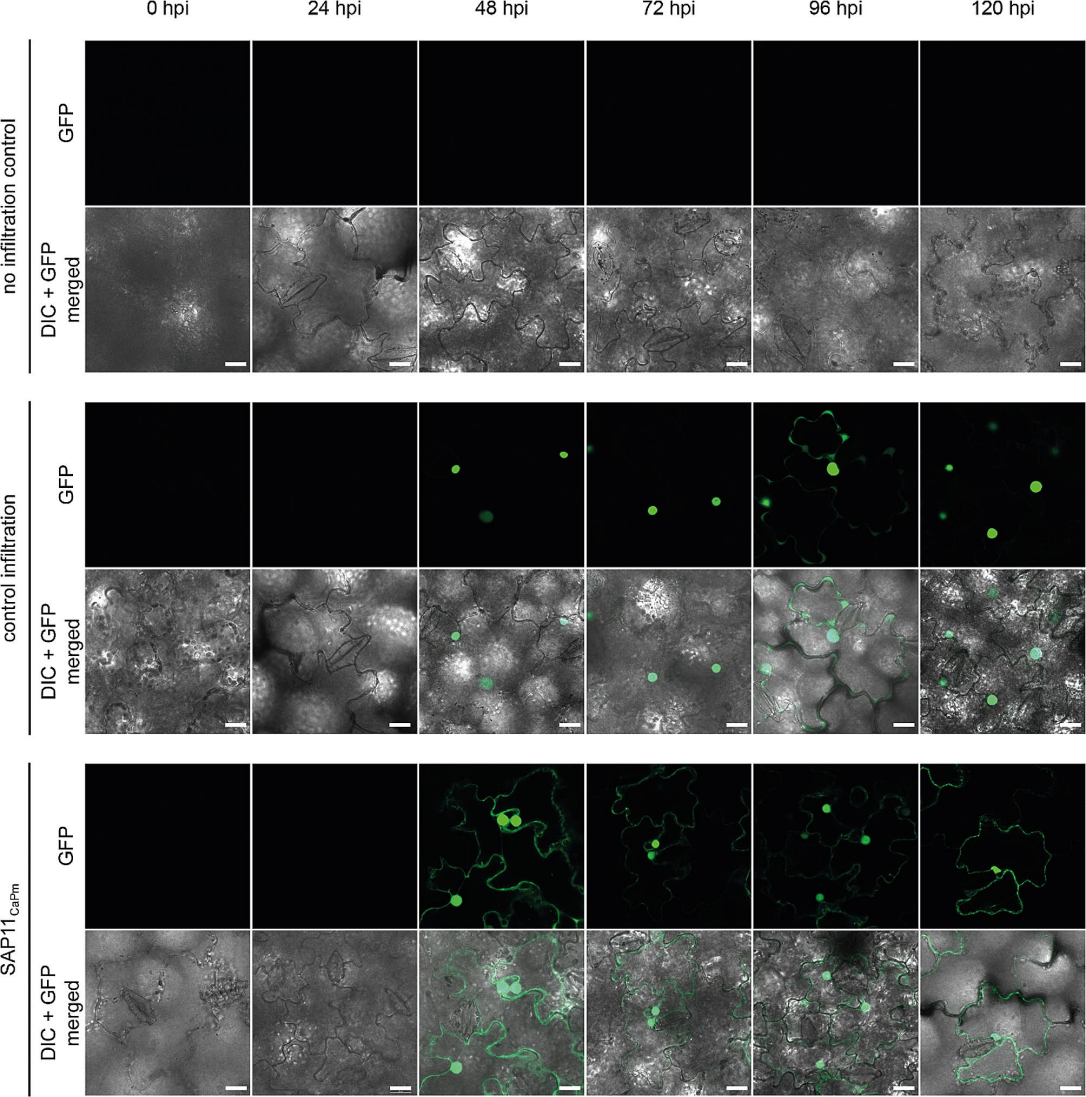
Detection of GFP-NLS (green fluorescent protein fused to a nuclear localization sequence, control infiltration) and SAP11_CaPm_:GFP (SAP11_CaPm_) in infiltrated N. occidentalis leaves. No infiltration control shows images of leaves that were not infiltrated. GFP fluorescence was visualized by confocal laser scanning microscopy. Bars represent 20 μm. GFP = only GFP channel, DIC + GFP = Differential interference contrast microscopy merged to the GFP channel

To verify the presence of SAP11_CaPm_ fused to GFP in *N. occidentalis* H.-M. Wheeler cells, leaves were examined using confocal laser scanning microscopy. The occurrence of SAP11_CaPm_:GFP as well as of GFP-NLS from the control-infiltrations became visible at 48 h after infiltration, thereby lagging behind the rise of transcripts (Fig. 2). SAP11_CaPm_:GFP was observed to localize to the cell nucleus and the cytoplasm of infiltrated *N. occidentalis* H.-M. Wheeler cells, while the GFP-NLS in control-infiltration localized only to the cell nucleus.

### De novo transcriptome assembly

A total of 54,704,261 (GC content: 43%) adapter and quality trimmed paired end reads from a pool of 51 RNA samples from *N. occidentalis* H.-M. Wheeler plants infiltrated or not were used for the de novo transcriptome assembly with Trinity v2.9. The clean reads were assembled, resulting in 166,787 transcripts, with an average length of 1,034 bp and an N_50_ of 1,504 bp.

The transcriptome was further decontaminated from sequences originating from species other than the order *Solanales* using the Model-based Categorical Sequence Clustering MCSC decontamination pipeline, that is based on the Model-based Categorical Sequence Clustering (MCSC) algorithm [30]. The decontaminated transcriptome contained 153,640 transcripts with an average length of 1,076 bp, N_50_ of 1,559 bp and a GC content of 39.57%.

This Transcriptome Shotgun Assembly project has been deposited at DDBJ/ENA/GenBank under the accession GKBG00000000. The version described in this paper is the first version, GKBG01000000.

The sequencing dataset used in this study is available in the NCBI repository with BioProject ID PRJNA871046.

### Differential expression analysis

**Fig. 3:**
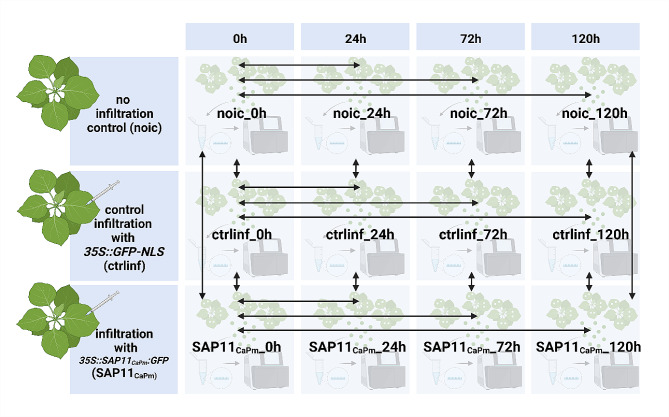
Identification of DETs in leaf samples of three different treatment groups. The first control group was not infiltrated (ni), the second control group was infiltrated with GFP-NLS under control of 35S promoter (ctrl) and the third group was infiltrated with 35S::SAP11_CaPm_:GFP (SAP11_CaPm_). Arrows show the comparisons that were made with DEseq2 [36]

The RNA-seq libraries obtained from leaf samples infiltrated with *A. tumefaciens* to express either *SAP11*_*CaPm*_:*GFP* or *GFP-NLS* or non-infiltrated were subjected to transcriptome analysis. To get insights into SAP11_CaPm_-mediated changes, differential expression analysis was done using DEseq2 [36] within the Trinity Package (v2.11.0) [27], making pairwise comparisons of non-infiltrated (ni), control-infiltrated (ctrl) and SAP11_CaPm_ infiltrated (SAP11_CaPm_) samples at different time points (Fig. 3).

**Fig. 4:**
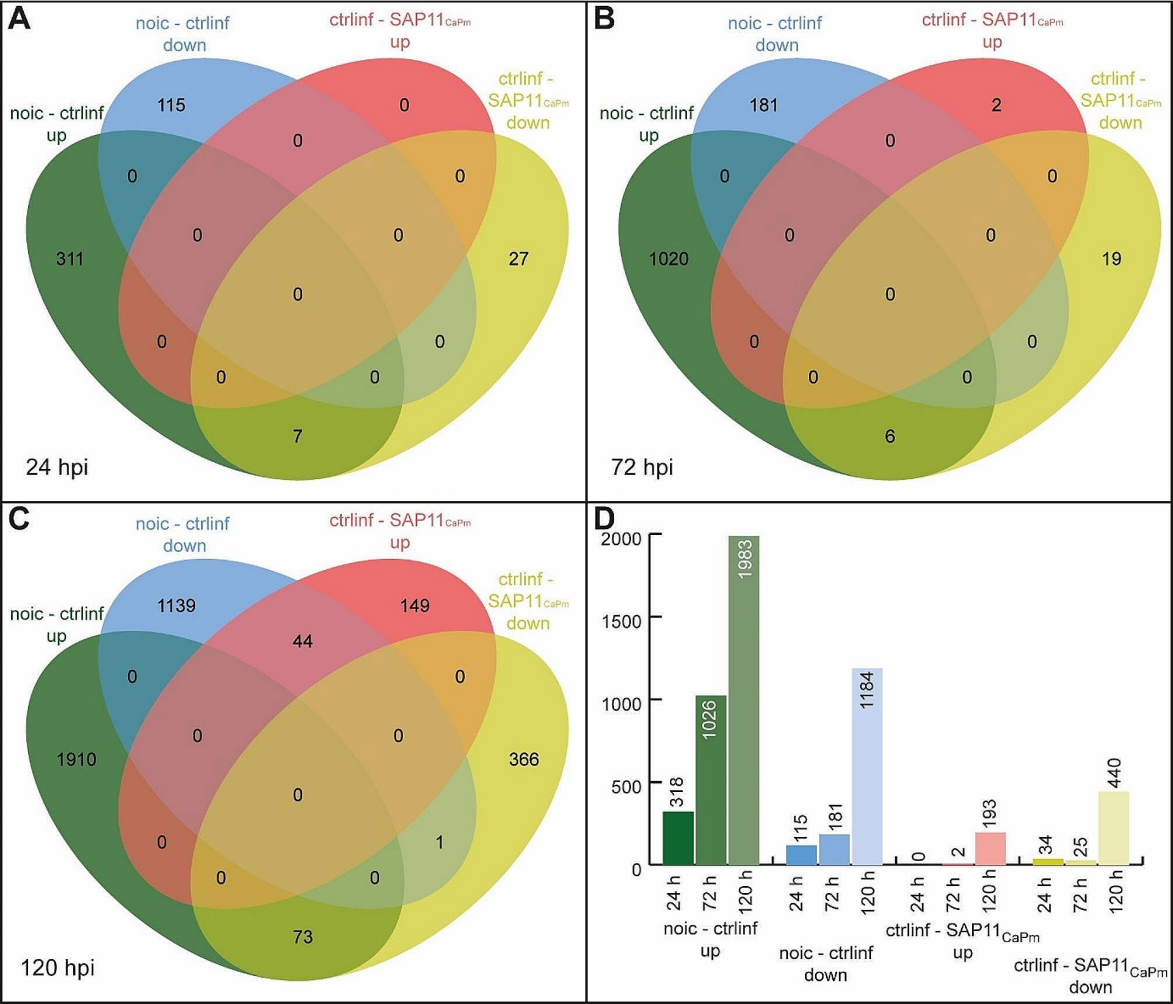
Numbers of DETs at log2fold change (L2FC) of ≥ ± 2; 4-fold differential expression, p-value cutoff for FDR < 0.001. The VENN diagram shows DETs detected at (**A**) 24, (**B**) 72 and (**C**) 120 h after infiltration. Up- and downregulated transcripts in control-infiltrated samples and SAP11_CaPm_ infiltrated samples were analyzed using jvenn [37]. (**D**) The barplot summarizes for each time point the total number of up- (green) and downregulated (blue) transcripts upon control- infiltration in comparison to non -infiltration and up- (red) or downregulated (yellow) transcripts due to infiltration with SAP11_CaPm_ in comparison to control-infiltration

Differentially expressed transcripts (DETs) were identified, which occurred over time within a treatment group or between treatment groups at the same time point (Fig. 4).

The first DETs in infiltrated leaves were detectable at 24 h after infiltration (Fig. 4A, D). In comparison to non-infiltrated leaves, control-infiltrated *N. occidentalis* H.-M. Wheeler leaves showed 318 upregulated and 115 downregulated transcripts. Only a few DETs were detectable between control-infiltrated and the SAP11_CaPm_-infiltrated samples: 34 transcripts were downregulated in SAP11_CaPm_ expressing samples compared to the control-infiltration. Seven of these 34 transcripts were upregulated in control-infiltrated samples compared to the non-infiltrated samples.

At 72 h after infiltration (Fig. 4B, D). a more substantial number of DETs were evident. Control-infiltrated leaves exhibited 1026 upregulated and 181 downregulated transcripts compared to non-infiltrated leaves. In contrast, SAP11_CaPm_-expressing leaves showed only two upregulated and 25 downregulated transcripts compared to control-infiltrated leaves. Six out of these 25 downregulated transcripts were upregulated in control-infiltrated leaves.

The highest number of DETs was observed at 120 h after infiltration (Fig. 4C, D).

Control-infiltrated samples had 1983 upregulated and 1184 downregulated transcripts compared to non-infiltrated leaves. The comparison between SAP11_CaPm_ infiltration and control-infiltration revealed that 193 transcripts were upregulated, and 440 transcripts were downregulated due to SAP11_CaPm_ infiltration. Among these 44 of the upregulated transcripts were downregulated between non-infiltrated samples and control-infiltration and 73 downregulated transcripts were upregulated in the control-infiltrated leaves (Fig. 4C).

Table 1 shows transcripts that resulted differentially expressed at two different timepoints upon SAP11_CaPm_ expression (compared to ctrl). Interestingly, a transcript encoding a protein modifier of *snc1,1* (MOS1) was downregulated 24 h after infiltration (L2FC -11.54) but upregulated 120 h after infiltration (L2FC 12.40). Three transcripts were downregulated at 24 h and 120 h, but only for one of them, i.e. the preprotein translocase subunit SCY1, information regarding its function is available. The acyl-CoA thioesterase 2 was downregulated at 24 h and 72 h after infiltration with SAP11_CaPm_ (L2FC − 11.38).

Six genes were downregulated at 72 h and 120 h after infiltration with SAP11_CaPm_ (Table 1) in contrast to control-infiltration. Among those genes, genes encoding the serine/threonine-protein phosphatase BSL1, the protein CHROMATIN REMODELING 8 (CHR8) and a NTRC-like thioredoxin reductase were detected. Despite the downregulation in SAP11_CaPm_ infiltrated samples after 72 h and 120 h, the NTRC-like thioredoxin reductase and the pre-mRNA-splicing factor prp12 and THO complex subunit 4D-like were upregulated upon infiltration (in the absence of SAP11_CaPm_): the NTRC-like thioredoxin reductase was upregulated after 72 h and 120 h, the pre-mRNA-splicing factor prp12 after 72 h and THO complex subunit 4D-like after 24 h and 72 h.

The putative DUF21 domain-containing protein At3g13070, as well as BSL1 and the pre-mRNA-splicing factor prp12 were not only downregulated in SAP11_CaPm_ infiltrated samples in comparison to control-infiltration but also in comparison to the not-infiltrated samples.

**Table 1:** Annotations of DETs, that are differentially regulated at two different time points (A and B) upon SAP11_CaPm_ expression (compared to ctrl)

Time point A	L2FC	Time point B	L2FC	Annotation
24 h	-12.35	120 h	-9.51	XM_019400596.1 PREDICTED: *Nicotiana attenuata* preprotein translocase subunit SCY1
24 h	-12.03	120 h	-9.31	XM_019375308.1 PREDICTED: *Nicotiana attenuata* putative DUF21 domain-containing protein At3g13070
24 h	-11.31	120 h	-10.25	XM_009759060.1 PREDICTED: *Nicotiana sylvestris* angio-associated migratory cell protein (LOC104210218)
24 h	-11.38	72 h	11.26	XM_016613005.1 PREDICTED: *Nicotiana tabacum* acyl-CoA thioesterase 2-like (LOC107791023)
24 h	-11.54	120 h	12.40	XM_009765541.1 PREDICTED: *Nicotiana sylvestris* protein MODIFIER OF SNC1 1 (LOC104215684)
72 h	-12.14	120 h	-10.69	XM_019370017.1 PREDICTED: *Nicotiana attenuata* serine/threonine-protein phosphatase BSL1 (LOC109207134)
72 h	-11.45	120 h	-10.19	XM_009610029.3 PREDICTED: *Nicotiana tomentosiformis* protein CHROMATIN REMODELING 8 (LOC104102344)
72 h	-3.49	120 h	-9.25	XM_009774985.1 PREDICTED: *Nicotiana sylvestris* dedicator of cytokinesis protein 7 (LOC104223519)
72 h	-12.11	120 h	-8.89	XM_016623444.1 PREDICTED: *Nicotiana tabacum* thioredoxin reductase NTRC-like (LOC107800295)
72 h	-2.38	120 h	-9.11	XM_019377213.1 PREDICTED: *Nicotiana attenuata* pre-mRNA-splicing factor prp12 (LOC109213418)
72 h	-12.23	120 h	-10.20	XM_016605885.1 PREDICTED: *Nicotiana tabacum* THO complex subunit 4D-like (LOC107784716)

### qPCR validation of selected DETs

A total of 15 transcripts, that were differentially expressed between control-infiltration and SAP11_CaPm_ infiltration, were selected as candidates for qPCR validation (Table 2).

**Table 2:** Selected transcripts for qPCR validation with annotation and L2FC changes

Sample A	Sample B	Accession Nr.	Description	L2FC
ctrl_120h	SAP11_CaPm__120h	XM_019373029.1	PREDICTED: *Nicotiana attenuata* calmodulin-binding receptor-like cytoplasmic kinase 3 (LOC109209712)	-2.3
ctrl_120h	SAP11_120h	XM_009804809.1	PREDICTED: *Nicotiana sylvestris* probable leucine-rich repeat receptor-like protein kinase At5g49770 (LOC104248540)	-9.5
ctrl_120h	SAP11_120h	XM_019390873.1	PREDICTED: *Nicotiana attenuata* proline dehydrogenase 2	13.3
ctrl_120h	SAP11_120h	XM_019378812.1	PREDICTED: *Nicotiana attenuata* protein PHYLLO	12.4
ctrl_120h	SAP11_120h	XM_019370442.1	PREDICTED: *Nicotiana attenuata* calmodulin-7 (LOC109207504)	-10.9
ctrl_120h	SAP11_120h	JF897607.1	*Nicotiana benthamiana* chloroplast PsbP1 precursor (psbP1) mRNA	-11.2
ni_120h	SAP11_120h	XM_016603442.1	PREDICTED: *Nicotiana tabacum* probable WRKY transcription factor 31 (LOC107782559)	12.6
ctrl_120h	SAP11_120h	XM_016603442.1	PREDICTED: *Nicotiana tabacum* probable WRKY transcription factor 31 (LOC107782559)	13.1
ctrl_120h	SAP11_120h	XM_019379586.1	PREDICTED: *Nicotiana attenuata* oxygen-evolving enhancer protein 1	-11.2
ctrl_120h	SAP11_120h	XM_019372421.1	PREDICTED: *Nicotiana attenuata* SKP1-like protein 21 (LOC109209196)	-8.9
ctrl_24h	SAP11_24h	XM_019403282.1	PREDICTED: Nicotiana XXXattenuate polyadenylate-binding protein-interacting protein 7 (LOC109237039)	-11.4
ctrl_120h	SAP11_120h	XM_016640507.1	PREDICTED: *Nicotiana tabacum* protein kinase APK1A	12.7
ctrl_72h	SAP11_72h	XM_016623416.1	PREDICTED: *Nicotiana tabacum* probable xyloglucan endotransglucosylase/hydrolase protein 6 (LOC107800268)	-2.8
ctrl_120h	SAP11_120h	XM_009778335.1	PREDICTED: *Nicotiana sylvestris* TMV resistance protein N-like (LOC104226353)	-10.7
ctrl_120h	SAP11_120h	XM_016646486.1	PREDICTED: *Nicotiana tabacum* protein LUTEIN DEFICIENT 5	-9.7
ctrl_120h	SAP11_120h	XM_016646486.1	PREDICTED: *Nicotiana tabacum* protein LUTEIN DEFICIENT 5	12.0
ctrl_120h	SAP11_120h	XM_019407249.1	PREDICTED: *Nicotiana attenuata* patatin-like protein 2 (LOC109240587)	-10.0

**Fig. 5:**
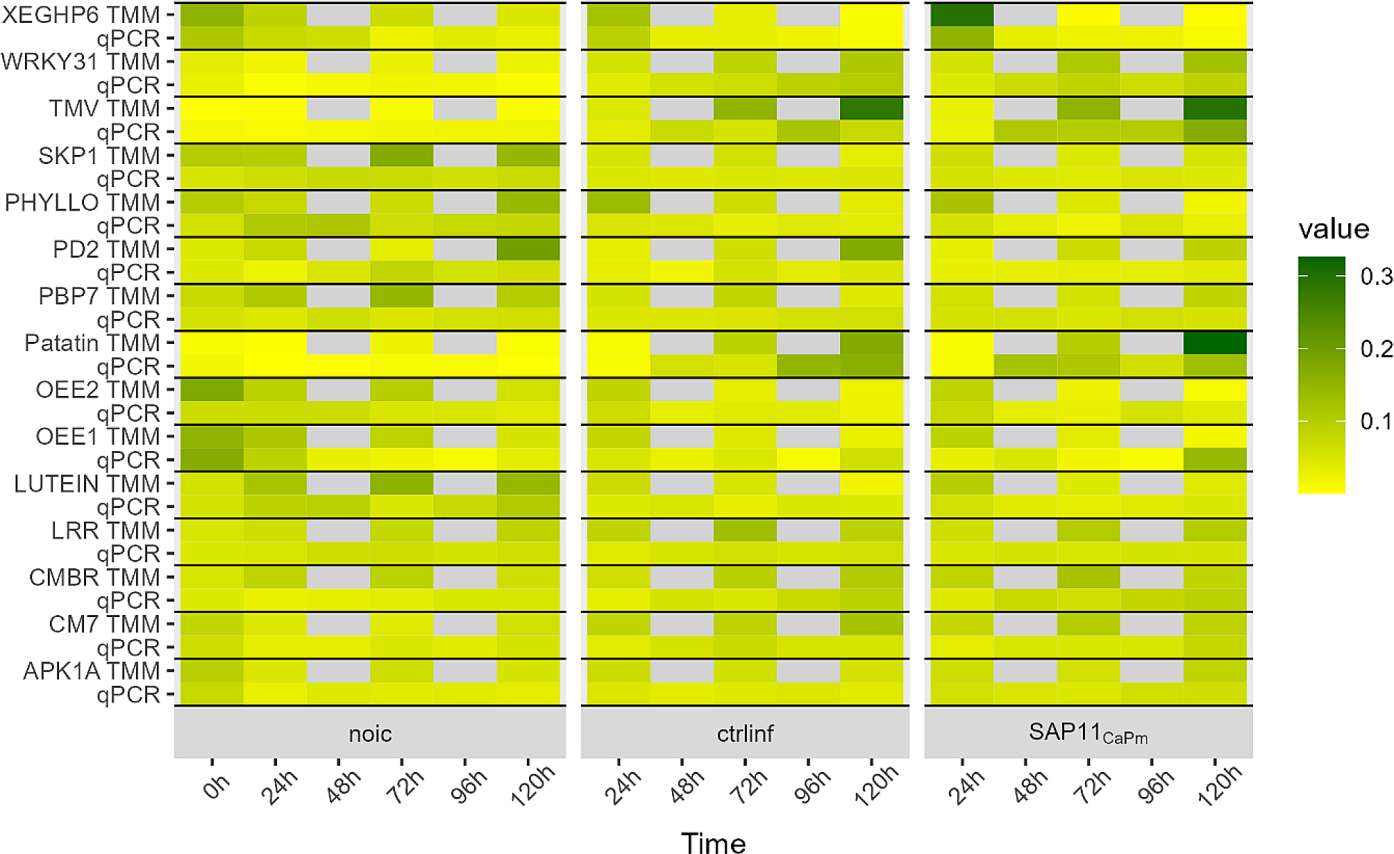
Heatmap of transcript accumulation as determined by RT-qPCR analysis and as TMM from RNA-seq data. Values are given as sum normalized values

RT-qPCR analyses from the 15 selected genes revealed high variance between biological replicates and no significant differences could be detected in the selected comparisons. The expression pattern, however, followed the same trend as those in RNA-seq data (Fig. 5). Taken together, these validation results of selected genes, the SAP11_CaPm_ expression verified by qPCR (Fig. 1) and the RNA-seq data obtained, provide novel insight into the early effects of SAP11_CaPm_ on the transcriptome of *N. occidentalis* H.-M. Wheeler.

### Gene ontology (GO) enrichment and analysis

**Fig. 6:**
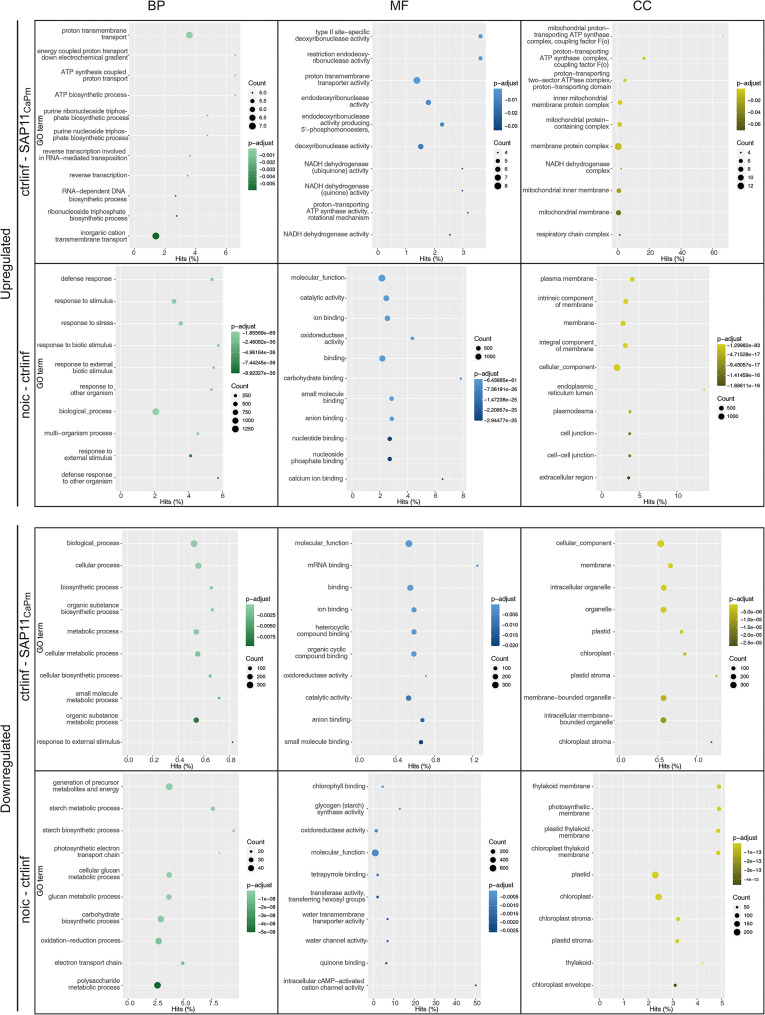
GO enrichment in samples taken 120 h after infiltration. The GO enrichment is separated by biological process (green), molecular function (blue) and cellular component (yellow). The y-axis represents the top 10 GO terms, while the x-axis displays the percentage of enriched GO terms within each category. The size of the filled circles corresponds to the number of transcripts associated with the GO term, and the color of the circles reflects the adjusted p-value

All groups of up- and downregulated DETs (Fig. 4) were analyzed using the Trinity v2.11.0 build in pipeline for GOseq [38]. The results were separated by the three sub-ontologies of GO: Molecular Function (MF), Cellular Component (CC) and Biological Process (BP) (Fig. 6). The datasets were further analyzed with the rrvgo package [41] which groups GO terms based on their semantic similarity and results were represented by a scatter plot (see Additional file 2).

After 24 h and 72 h no significant (FDR < 0.5) enriched GO terms were found in the group of SAP11_CaPm_ downregulated transcripts in comparison to control-infiltration. The two upregulated transcripts in SAP11_CaPm_ expressing samples at 72 h were assigned to the term “regulation of protein metabolic process” (BP). Enriched terms in the group of downregulated transcripts in SAP11_CaPm_ expressing leaves compared to control-infiltration at 120 h comprised transcripts that were categorized into “cellular process”, “biosynthetic process” or “response to external stimulus” (Fig. 6). Enriched GO terms in MF were mainly related to binding processes, such as “mRNA binding”, “organic cyclic compound binding” or “magnesium chelatase activity”, whereas CC enriched terms were “plastid”, “chloroplast” or “membrane-bounded organelle” (Fig. 6). Employing the rrvgo analysis within the set of 366 transcripts (Fig. 4; ctrl-SAP11_CaPm_ down) unaffected by infiltration but downregulated during SAP11_CaPm_ expression unveiled a cluster associated with both “defence response” and “response to external stimulus”. The group of 149 upregulated transcripts (Fig. 3) in SAP11_CaPm_ expressing samples after 120 h compared to the control-infiltration mainly contained transcripts that were assigned to BP GO terms like “proton transmembrane transport”, “reverse transcription involved in RNA − mediated transposition” and “ATP biosynthetic process”. The enriched MF GO terms were related to “endodeoxyribonuclease activity” and the enriched CC GO terms were mainly allocated to “mitochondrial protein − containing complex” and “proton − transporting ATP synthase complex, coupling factor F(o)”. The rrvgo analysis of all 193 upregulated transcripts at 120 h showed a cluster assigned to “ATP biosynthetic process” and “proton transmembrane transport” (Additional file 2).

In the group of upregulated transcripts found only in the control-infiltrated samples at 24 h, 72 h and 120 h after infiltration, GO terms for the category BP are enriched such as “defence response”, “response to biotic stimulus” or “response to stress. Downregulated transcripts after 24 h were enriched in BP GO terms related to “homeostasis”, while 72 h after infiltration the enriched BP terms were assigned to “photosynthesis, light harvesting”, “protein − chromophore linkage” or “electron transport chain”. At 120 h after infiltration the most enriched BP terms were “starch metabolic process”, “cation transport” and “photosynthetic electron transport chain” (Fig. 6). The rrvgo analysis revealed a cluster of transcripts related to “ATP biosynthetic process” that is, in contrast to infiltration with SAP11_CaPm_ (ctrl - SAP11_CaPm_), upregulated upon control-infiltration (Additional file 2). For both timepoints, 72 h and 120 h most of the enriched CC terms are chloroplast related (“thylakoid membrane”, “photosystem”, “chloroplast”).

### Functional analysis of groups

**Fig. 7:**
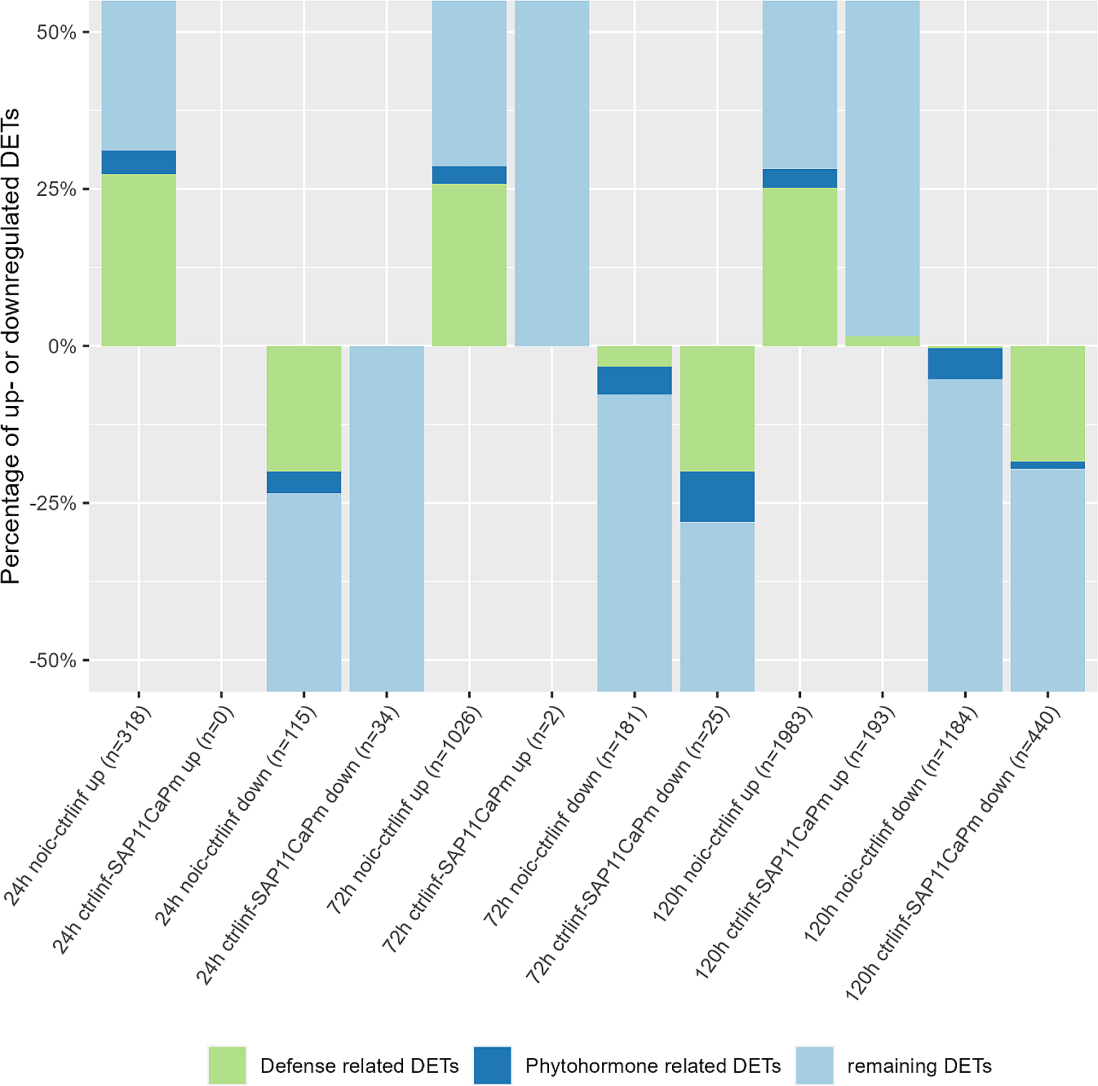
Analysis of GO annotations. Percentage of defense and phytohormone related differentially expressed transcripts, according to GO annotation

The GO annotations were selectively refined to include transcripts associated with defense or stress terms and phytohormone-related terms. As depicted in Fig. 7, the distribution of defense/stress and phytohormone-related terms in single DET groups is illustrated. Over 25% of the upregulated transcripts in the control-infiltrated samples were identified as defense or stress-related. Notably, in the downregulated DETs of control-infiltrated samples, the proportion of defense/stress-related transcripts decreases over time. In the group of upregulated DETs in SAP11_CaPm_-infiltrated leaves compared to control-infiltrated samples, almost no defense/stress or phytohormone-related transcripts were observed after 72 h (0%) or 120 h (0.5%). Conversely, in the downregulated transcripts of the same group, 28% of the transcripts (72 h) and 19.5% (120 h) are associated with defense/stress or phytohormone-related terms. The Additional file 3 lists all transcripts that were assigned to defense/stress or phytohormone-related terms.

### Network analysis

A network analysis of the different groups of DETs was performed using STRING [42].

**Fig. 8:**
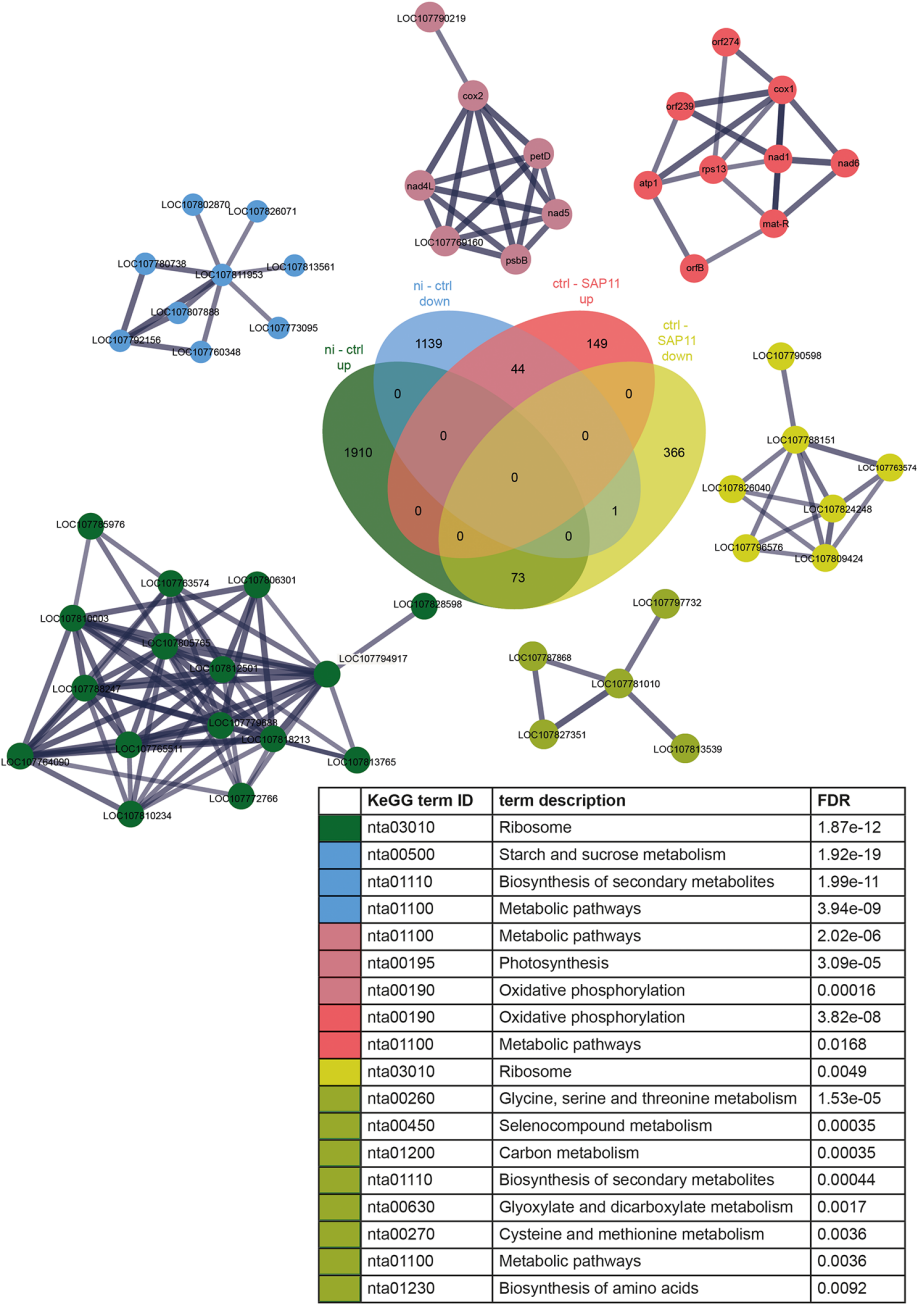
Network analysis of up- and downregulated transcripts. STRING analysis was followed by MCL (Markov Cluster Algorithm) clustering. The main network clusters within each group with their KEGG annotations (see coloring) are shown

It showed that KEGG pathways related to ribosome was upregulated upon infiltration (Fig. 8, ni – ctrl up), while the starch and sucrose metabolism was downregulated due to the infiltration process itself (Fig. 8, ni – ctrl down).

The main network cluster in the group of downregulated transcripts upon SAP11_CaPm_ infiltration (ctrl vs. SAP11_CaPm_) showed enriched KEGG pathways related to ribosome, while the main cluster of up regulated transcripts is enriched in oxidative phosphorylation and metabolic pathways. Detailed information about KEGG enriched transcripts and annotation of the main network cluster transcripts can be found in Additional file 4 and Additional file 5.

## Discussion

Pathogen-derived effectors are important players in the process by which a pathogen modulates its host’s metabolism. To get insights into the role of SAP11_CaPm_, an effector produced by ‘*Ca*. P. mali’, the causal agent of apple proliferation disease, early changes in gene expression upon expression of SAP11_CaPm_ were analyzed by infiltration RNA-seq [20]. For this, *SAP11*_*CaPm*_ was transiently expressed by agroinfiltration in *N. occidentalis* H.-M. Wheeler leaves, since it has been shown that this model plant can be infected with ‘*Ca*. P. mali’ [10, 19, 21–23]. Most importantly, transiently expressed SAP11_CaPm_ fused to GFP was detected in the nucleus of leaf cells, thereby occurring in the same subcellular compartment in which transcription factors occur to manipulate gene expression. This location is thus important evidence that *N. occidentalis* gene expression might be actively altered by SAP11_CaPm_. A reference genome of *N. occidentalis* H.-M. Wheeler is, however, not available, thus we opted for an approach where the *N. occidentalis* H.-M. Wheeler transcriptome was assembled de novo and used as a reference for the differential expression analysis.

A major problem with de novo assembling are biological contaminations of samples [30, 49]. These contaminations might derive from bacteria residing on the plant’s surface or be of human origin [50]. To filter and exclude these contaminations from our de novo assembled *N. occidentalis* H.-M. Wheeler transcriptome we used a pipeline based on the MCSC algorithm [30].

The transcript levels of *SAP11*_*CaPm*_ increased continuously until 96 h after infiltration, while it decreased at 120 h after infiltration. This result was slightly different compared to a second independent experiment, in which the expression remained stable and unchanged after 48 h and until 120 h after infiltration. Those differences in *SAP11*_*CaPm*_ expression strength might affect the putative changes in host’s gene expression profiles, although the protein was detectable to almost similar levels up to 120 h after infiltration. A group of differentially expressed transcripts in RNAseq was selected for the validation of their expression by RT-qPCR. Some of the qPCR results were very variable between biological replicates. Even though obtained from the same experimental setup, independent biological samples can inherently vary in their gene expression due to differences in cellular heterogeneity, or other factors. This inherent variability between biological replicates is a common source of variation in transcriptome analyses [51]. Nevertheless, the comparison of RNA-seq data with qPCR data of selected genes from the independent leaf samples, showed a common trend in both data sets. This corroborates findings derived from RNA-seq data by an independent method. In infected apple trees, the natural host plant, *SAP11*_*CaPm*_ expression is not stable throughout the year [16] and the degree of colonization by phytoplasma, plant growth and climatic conditions might influence the spatio-temporal expression of the effector. Nonetheless, using another natural host of ‘*Ca*. P. mali’, allowing transient expression assays, helps to unravel the transcriptional changes in the plant due to the action of one single effector protein. The highest number of DETs was detected at 120 h after infiltration. Interestingly, six genes were commonly downregulated at 72 h and 120 h after infiltration; four of these transcripts were allocated to defense-related terms in the GO annotation. Among them, the genes encoding the protein phosphatase BSL1 and chromatin remodeling8 (CHR8) were identified. The protein phosphatase BSL1 belongs to the BSU1 family and contributes to the brassinosteroid signalling. BSL1 interacts with the *Phytophtora infestans* effector protein PiAVR2 and acts as a susceptibility factor by affecting the balance between growth and immunity in plants [52–54].

CHR8 belongs to the switch2/sucrose non-fermenting2 (SWI2/SNF2) chromatin remodeling gene family that is involved in DNA damage response (Shaked et al. 2006). It has been shown that CHR8 is upregulated during an artificial infection of *A. thaliana* with cabbage leaf curl virus [55] as well as during genotoxic stress [56], indicating its potential role in plant stress response.

The gene encoding the modifier of snc1,1 (MOS1) was downregulated at 24 h and upregulated at 120 h after *SAP11*_*CaPm*_ infiltration. MOS1 regulates the nucleotide binding site-Leu-rich repeat (NB-LRR) type R protein SNC1 [57]. MOS1 interacts with AtTCP15, while AtTCP15 directly binds to SNC1 and thereby modulates the plant immune response [58]. Due to that binding, MOS1 enhances the activity of SNC1 and helps to reinforce the defense response. Based on the results of this study it can be assumed that the MOS1 triggered immune response is suppressed in the early stage of SAP11_CaPm_ expression, while it recovers later.

Our results show that the occurrence of SAP11_CaPm_ led to a downregulation of plant defense (Fig. 7). Nonetheless, it should be noted that the infiltration process itself induces several transcriptional changes within the infiltrated leaf area [59, 60]. Agroinfiltration induces host defense and alters phytohormone levels [59, 61, 62]. This is in line with the findings in Fig. 7. Transcripts related to plant defense and phytohormones are upregulated in control-infiltrated leaves. However, when comparing control-infiltration to SAP11_CaPm_ infiltration, it is evident that defense and phytohormone related transcripts are downregulated. The regulation of phytohormones is key to several phytoplasma diseases and plays a role in the regulation of plant defence responses against phytoplasmas [63]. The observed downregulation observed in this study of phytohormone transcripts thus indicates that SAP11_CaPm_ is able to suppress plant defense response.

At the same time, however, the ATP biosynthetic processes are upregulated. Phytoplasmas are highly dependent on metabolic compounds from their hosts since they lack different metabolic genes, among them genes for ATP synthase, glycolysis and for oxidative phosphorylation [64–67]. In the same line, the ATP biosynthetic process and the oxidative phosphorylation are upregulated upon SAP11_CaPm_ expression in *N. occidentalis* H.-M. Wheeler. This might reflect the enhanced levels of glycerolipid and glycerophospholipid metabolites and the numerous differentially expressed carbohydrate metabolism genes shown for sweet cherry virescence (SCV) phytoplasma infected sweet cherry trees [68, 69].

It can be assumed that an increase of the expression of genes involved in energy production in the host plant helps the phytoplasma to obtain sufficient metabolites and nutrients necessary for proliferation and colonization.

In contrast to metabolic genes, transcripts assigned to the chloroplast were downregulated 120 h upon SAP11_CaPm_ infiltration. Photosynthesis rates have been shown to be reduced in many different phytoplasma infections [70–72]. In line with that, several transcriptomic and proteomic studies of different phytoplasma infected plants have shown that numerous genes involved in photosynthesis are downregulated during infection [73]. The finding that SCY1 is strongly downregulated after 24 h and consistently at 120 h opens new insights on the possible SAP11 impact on chloroplast. SCY1 is involved in preprotein localization to the thylakoid [74] and mutant plants for SCY1 show impairment in thylakoid biogenesis [75] with a chloroplast-to-nucleus retrograde signal whit impaired production of nuclear-encoded chloroplast proteins [76] and chlorotic phenotypes [77]. During periods of darkness, SCY1 levels increase in the chloroplast which augments the import of nuclear-encoded proteins [78]. .

It is still unknown whether phytoplasma effector proteins directly target the chloroplast or if photosynthesis is compromised due to the changed plant metabolism [79]. A potential role of SAP11 in reducing the activity of photosystem II (PSII) by binding the AtTCP13 transcription factor has been suggested [79]. AtTCP13 is also known as PTF1, a transcription factor that regulates gene expression in chloroplasts via the plastid-encoded polymerase PEP [80]. PEP is the major chloroplast transcriptase and regulates the expression of *psbD* encoding the reaction center protein D2 of PSII. Since in *A. thaliana ptf1* mutants the expression of *psbD* is reduced [81], it can be assumed that SAP11 binding to TCP13 has a similar effect.Even though *psbD* was not downregulated in this study, two other genes encoding proteins from PSII were downregulated upon SAP11_CaPm_ expression, namely the oxygen-evolving enhancer proteins 1 (OEE1/PsbO) and OEE2 (PsbP). Interestingly, OEE2/PsbP is directly targeted by a *Plasmopara viticola* RXLR effector protein [82, 83], while OEE1/PsbO binds to HIPM (HrpN-interacting protein from *Malus* spp.), a susceptibility gene for *Erwinia amylovora* infection in apple [84]. *Nicotiana benthamiana* Domin *psbP* knockout mutants showed a reduced growth and bleached leaves and were less susceptible to *Phytophtora capsici* infection [82]. In the same line, transgenic grapevine lines overexpressing *psbP* were more susceptible to *P. viticola* infection [82], indicating that PsbP downregulation enhances immunity. In contrast OEE1/PsbO and OEE2/PsbP proteins were more abundant in leaves of powdery mildew resistant cucumbers than in susceptible ones [85].

## Conclusions

Our findings revealed a downregulation of defense-related genes, suggesting a suppression of the plant’s immune response by *SAP11*_*CaPm*_. Moreover, the modulation of genes involved in oxidative phosphorylation, as well as upregulation of ATP biosynthetic processes, hinted at a potential strategy employed by the phytoplasma via its effectors to exploit host metabolic pathways for its proliferation and colonization.

Additionally, the downregulation of transcripts related to chloroplast, such as SCY1, OEE1/PsbO or OEE2/PsbP suggest a potential link between SAP11_CaPm_ and the compromise of photosynthetic processes, possibly through interactions with chloroplast-related factors.

Our study contributes to the understanding of SAP11_CaPm_’s impact on phytoplasma host plants. However, questions regarding the direct targeting of chloroplasts and the intricate mechanisms leading to photosynthesis reduction and the effect on ATP biosynthetic processes and oxidative phosphorylation remain open. Future research elucidating these aspects will advance our understanding of phytoplasma-plant interactions and will result in new strategies for mitigating the impact of phytoplasma infections in agricultural settings.

### Electronic supplementary material

Below is the link to the electronic supplementary material.

Supplementary Material 1

Supplementary Material 2

Supplementary Material 3

Supplementary Material 4

Supplementary Material 5

## Data Availability

The datasets generated and/or analyzed during the current study are available in the NCBI repository with BioProject ID PRJNA871046 and contains 13 BioSample datasets (https://www.ncbi.nlm.nih.gov/bioproject/?term=PRJNA871046). The Transcriptome Shotgun Assembly project has been deposited at DDBJ/ENA/GenBank under the accession GKBG00000000 (https://www.ncbi.nlm.nih.gov/nuccore/2428580569).

## Abbreviations

ABA: Abscisic Acid
ATP: Adenosine Triphosphate
BP: Biological Process
BSL1: Serine/Threonine-protein phosphatase BSL1, BSU1-like 1
BSU1: BRI1 SUPPRESSOR 1
'*Ca*. P. mali’: ‘*Candidatus* Phytoplasma mali’
CaMV 35S: Cauliflower Mosaic Virus 35 S promoter
CC: Cellular Component
cDNA: Complementary DNA
CHR8: Chromatin Remodeling 8
DET: Differentially Expressed Transcript
DUF21: Domain of Unknown Function 21
ELI3-1: ELICITOR-ACTIVATED GENE3-1
FDR: False Discovery Rate
GC: Guanine-Cytosine (content in DNA)
GFP: Green Fluorescent Protein
GO: Gene Ontology
JA: Jasmonate
KEGG: Kyoto Encyclopedia of Genes and Genomes
L2FC: Log2 Fold Change
LOX2: LIPOXYGENASE2
MF: Molecular Function
MOS1: Modifier of snc1,1
NADPH: Nicotinamide Adenine Dinucleotide Phosphate
NB-LRR: Nucleotide-Binding Leucine-Rich Repeat
NTRC: NADPH-dependent thioredoxin reductase C
nt: Nucleotide collection database
OEE1/PsbO: Oxygen-Evolving Enhancer Protein 1
OEE2/PsbP: Oxygen-Evolving Enhancer Protein 2
PEP: Plastid-Encoded Polymerase
PiAVR2: *Phytophthora infestans* effector protein AVR2
PR1: PATHOGENESIS-RELATED GENE1
qPCR: Quantitative Polymerase Chain Reaction
RNA-seq: RNA sequencing
RBCS: Ribulose-1,5-bisphosphate carboxylase/oxygenase small subunit
RT-qPCR: Reverse Transcription Quantitative Polymerase Chain Reaction
SA: Salicylic acid
SCV: Sweet Cherry Virescence phytoplasma
SEM: Standard Error of the Mean
SWI2/SNF2: SWITCH2/SUCROSE NON-FERMENTING2
TCP: TEOSINTE BRANCHED1, CYCLOIDEA, and PROLIFERATING CELL FACTORS
TMM: Trimmed Mean of M values
tRBCS: Ribulose-1,5-bisphosphate carboxylase/oxygenase terminator
