# Supplementary material for: ‘Candidatus Phytoplasma mali’ SAP11-Like protein modulates expression of genes involved in energy production, photosynthesis, and defense in Nicotiana occidentalis leaves
Source: BMC Plant Biol. 2024 May 13;24:393. doi: 10.1186/s12870-024-05087-4 (PMC11089699; doi:10.1186/s12870-024-05087-4)
Supplement: Supplementary file 3 — Supplementary Material 3 [file 12870_2024_5087_MOESM3_ESM.pdf]

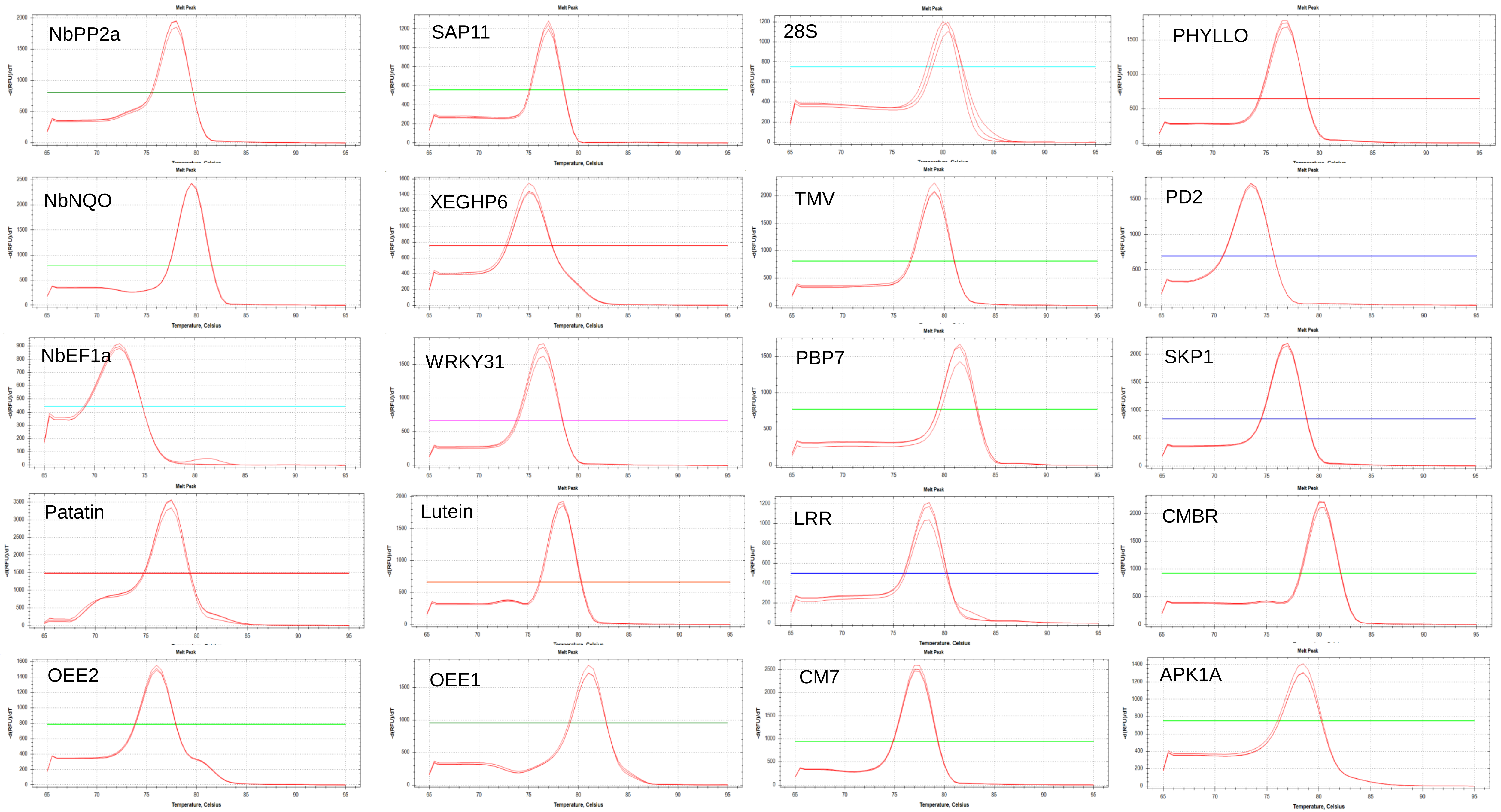

Additional file 1: Exemplary melting curve of amplicons generated by SYBR green quantitative PCR in this study.
