# Supplementary figures and images for: ‘Candidatus Phytoplasma mali’ SAP11-Like protein modulates expression of genes involved in energy production, photosynthesis, and defense in Nicotiana occidentalis leaves
Source: BMC Plant Biol. 2024 May 13;24:393. doi: 10.1186/s12870-024-05087-4 (PMC11089699; doi:10.1186/s12870-024-05087-4)

BP

MF

CC

ctrlinf - SAP11<sup>CaPm</sup>

noic - ctrlinf

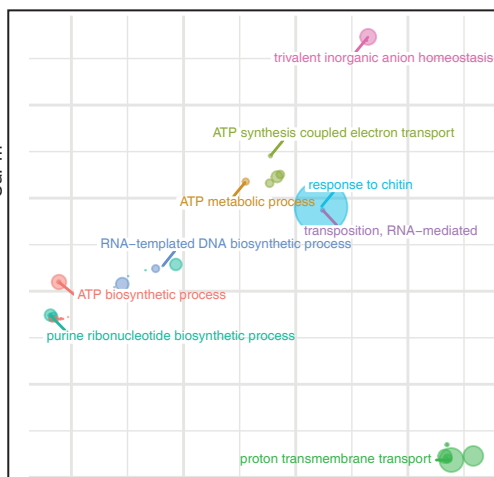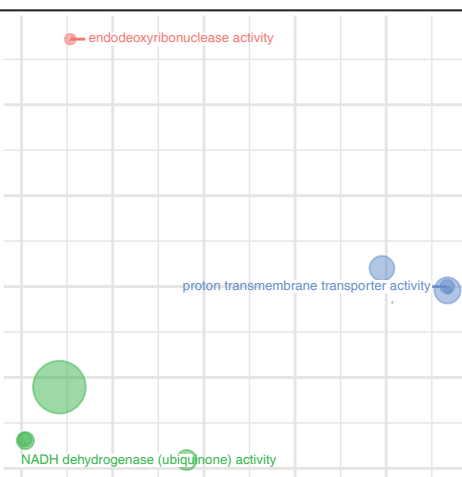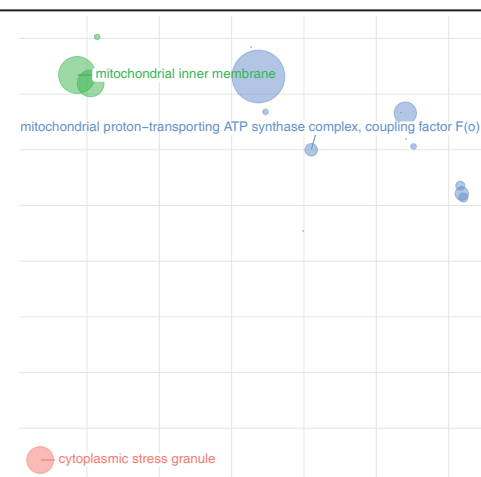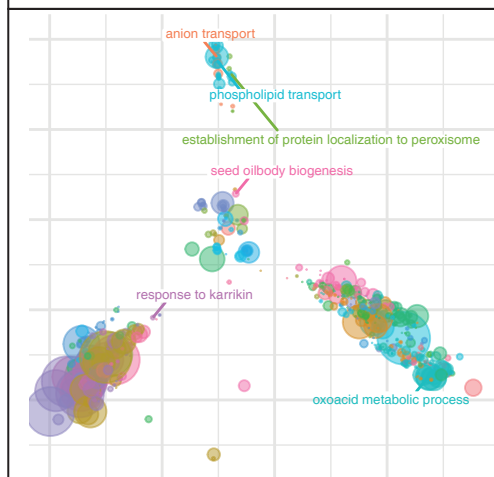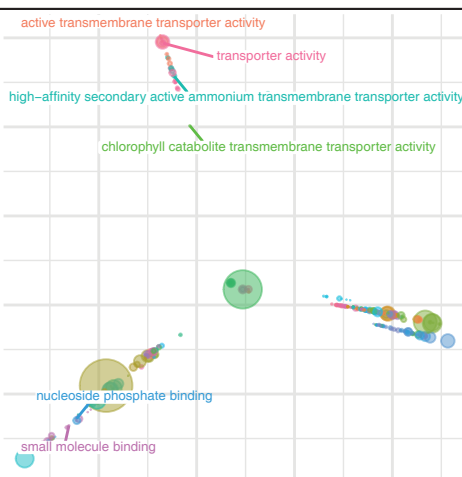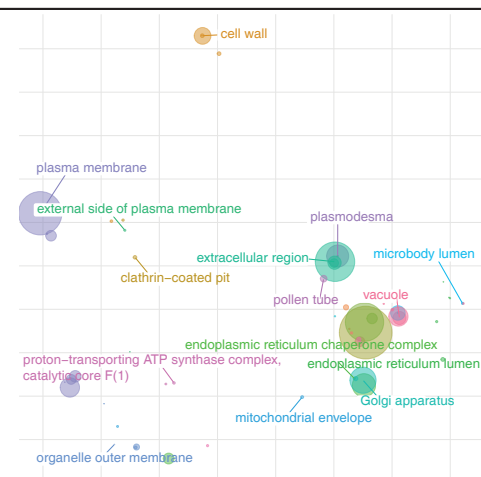ctrlinf - SAP11<sup>CaPm</sup>

noic - ctrlinf

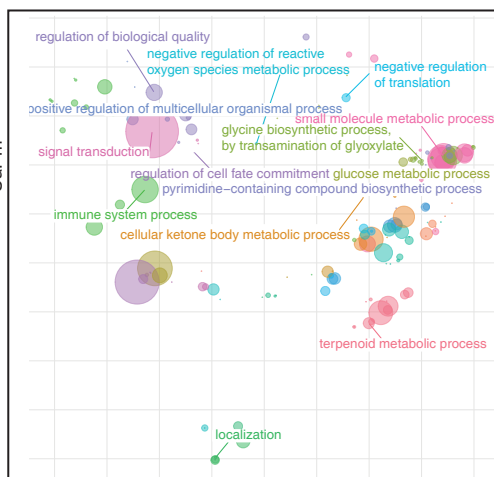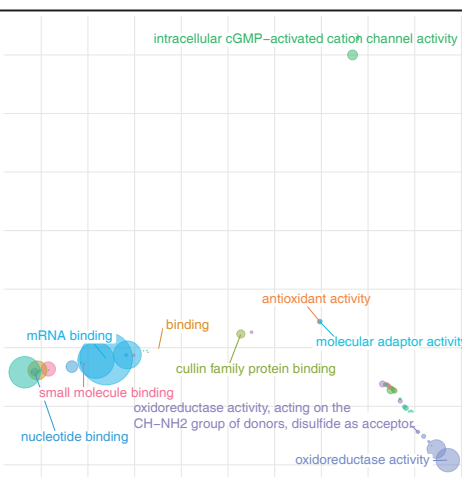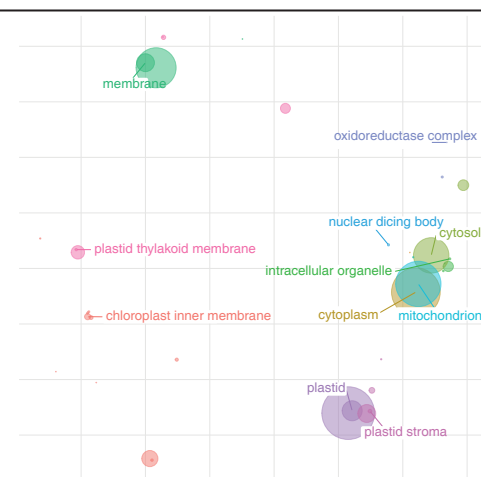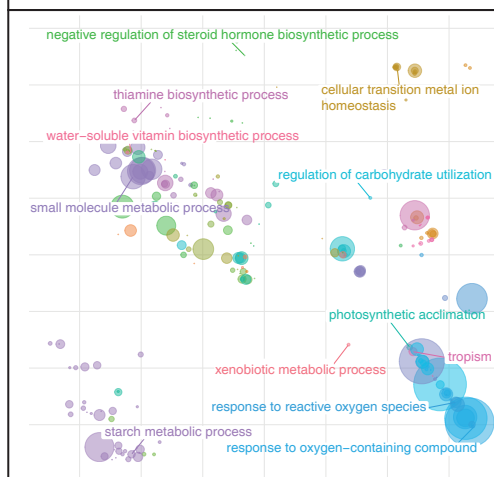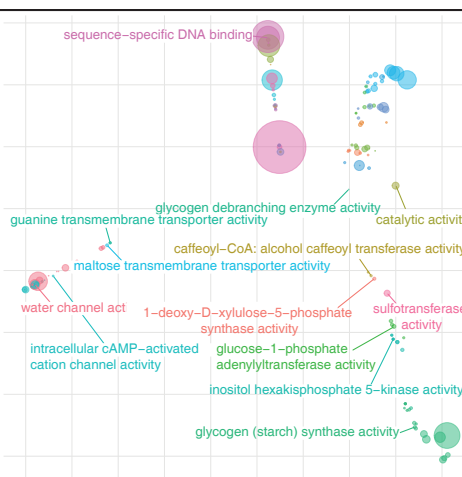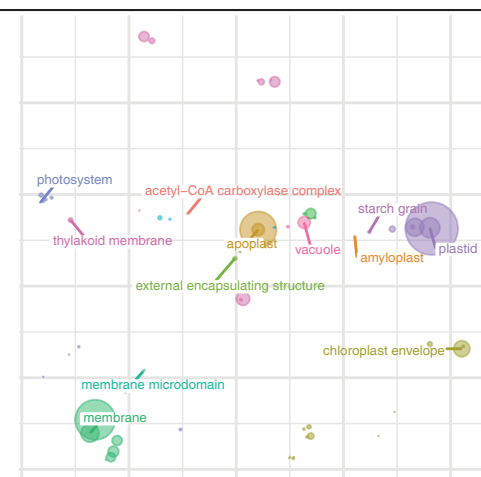

Supplement: Supplementary file 5 — Supplementary Material 5 [file 12870_2024_5087_MOESM5_ESM.pdf]
